# Correlation between Sleep Quality and Frailty Status among Middle-Aged and Older Taiwanese People: A Community-Based, Cross-Sectional Study

**DOI:** 10.3390/ijerph17249457

**Published:** 2020-12-17

**Authors:** An-Chen Shih, Lee-Hwa Chen, Chin-Chueh Tsai, Jau-Yuan Chen

**Affiliations:** 1 Department of Family Medicine, Chang Gung Memorial Hospital, Linkou Branch, Taoyuan 333, Taiwan; cooker791022@gmail.com; 2 Department of Athletic Training and Health, National Taiwan Sport University, Taoyuan 333, Taiwan; lhchen@ntsu.edu.tw (L.-H.C.); cctsai@ntsu.edu.tw (C.-C.T.); 3 Department of Medicine, College of Medicine, Chang Gung University, Taoyuan 333, Taiwan

**Keywords:** frailty, community-based, sleep quality, middle-aged and older adults

## Abstract

Poor sleep quality and frailty are common problems among aged people. However, the association between sleep quality and frailty in middle-aged and older people is seldom discussed in Asia, especially in Taiwan. This study investigated this association hopefully to provide pertinent knowledge for the prevention of frailty. We conducted a cross-sectional study and enrolled 828 subjects, 237 male and 591 female, aged 50–85 years old, from a community in Northern Taiwan. Poor sleep quality was defined as the Chinese version of the Pittsburgh Sleep Quality Index (CPSQI) > 5. Prefrailty and frailty were defined as fulfillment of one or two and three, respectively, of five phenotypic criteria: exhaustion, weakness, slowness, weight loss, and low physical activity. Our univariate analysis showed that the incidence of prefrailty/frailty in the group of poor sleep quality was higher than that in the group of CPSQI ≤ 5 (*p* < 0.001). Further multiple logistic regression analysis revealed that poor sleep quality was an independent factor for prefrailty and frailty status (odds ratio = 1.95, 95% confidence interval = 1.38–2.77), after adjustment for confounding factors. We concluded that poor sleep quality is independently associated with prefrailty and frailty status in our study population.

## 1. Introduction

Population aging, which describes a rise in life expectancy and a fall in birth rates, constitutes a major problem worldwide, particularly in Taiwan. According to data from the National Development Council, Taiwan became an aging society in 1993; then, it became an aged society in 2018, and it is projected to become a superaged society in 2025 [1]. Taiwan’s population is aging at an alarming rate, with only eight years to advance from the “aged society” stage to the “super-aged society” stage, which is much faster than the 11 years for Japan, 14 years for the U.S., 29 years for France, and 51 years for the UK [2]. People aged 65 years and older in Taiwan were estimated to represent 16% of the overall population in 2020. The costs resulting from the high proportion of elderly people in Taiwan’s population are considered a heavy burden on the national health insurance system [3]. Thus, population aging is a major social challenge.

Poor sleep, a common health problem, has a high incidence among elderly people [4,5]. Even in healthy people, destruction of the sleep cycle may lead to a greater response to stress, an increase in painful musculoskeletal sensitivity, and a decrease the quality of life. It may cause multi-comorbidities including cardiovascular and metabolic diseases in the long term [6]. Poor sleep quality is associated with the risk of hypertension and adverse cardio-metabolic effects [7]. Furthermore, sleep disorders such as obstructive sleep apnea were reported to be a major risk factor for psychiatric, cardiovascular, metabolic, or hormonal co-morbidity and mortality [7]. Sleep problems are associated with a range of adverse health outcomes [8], such as depression, anxiety, chronic pain, cardiovascular diseases (CVDs), metabolic diseases, cognitive impairment, physical disability, and even mortality [7,9]. These problems result in an economic burden exceeding $40 million annually in Taiwan [10].

Frailty is defined as an aging-related syndrome of physiological decline, which is characterized by marked vulnerability to adverse health outcomes including loss of function, loss of physiologic reserve, and increased vulnerability to disease and death [11]. Frailty is prevalent in elderly people and increases the risk of falls, disability, hospitalization, and mortality. Studies in Taiwan showed the prevalence of frailty and prefrailty were 6.8% and 40.5%, respectively [12]. Research reported that the prevalence estimates are 31.3–45.8% for prefrailty and 10.4–37.0% for frailty among community-dwelling elderly people [13]. In comparison, the prevalence of frailty in Taiwan was relatively lower, but the prevalence of prefrailty was above the average. The incidence of frailty and prefrailty was estimated to be 43.4 and 150.6 new cases per 1000 person-years worldwide, respectively [14]. The incidence of frailty was significantly higher in prefrail individuals than robust individuals [14]. Frailty among elderly people is identified through the presence of sarcopenia, reduced activity, poor appetite, osteoporosis, easy fatigability, frequent falls, and poor general health. Many medical conditions can cause fatigue, including cardiopulmonary, endocrinological or metabolic, hematologic or neoplastic, and psychological diseases as well as other reversible problems. Sleep disturbance is a problem that causes fatigue [15].

Although the association between frailty and poor sleep quality has been reported [16,17], two important issues may be raised. Firstly, the population of previous studies enrolled people mainly over 65 years old with some over 60 years old. The middle-aged population was seldom discussed about the relationship between sleep quality and frailty/prefrailty status. Recent large-scale studies have advocated that efforts to identify, manage, and prevent frailty should include middle-aged individuals, particularly those with multimorbidity [18]. Secondly, as mentioned above, there is a relatively higher prevalence rate of prefrailty in Taiwan, and the incidence of frailty was significantly higher in prefrail people. Thus, prevention from becoming frail or attaining prefrailty status is an important issue in Taiwan. This issue also deserves attention, given the fact that sleep problems are commonly found in middle-aged and older Taiwanese. Thus, the main aim of this study was to investigate the association between sleep quality and the risk of frailty among community-dwelling middle-aged and older adults Taiwanese people. Our findings hopefully may provide valuable information for the prevention of frailty by improving sleep problems in this population.

## 2. Materials and Methods

### 2.1. Study Design and Participants

**Figure 1:**
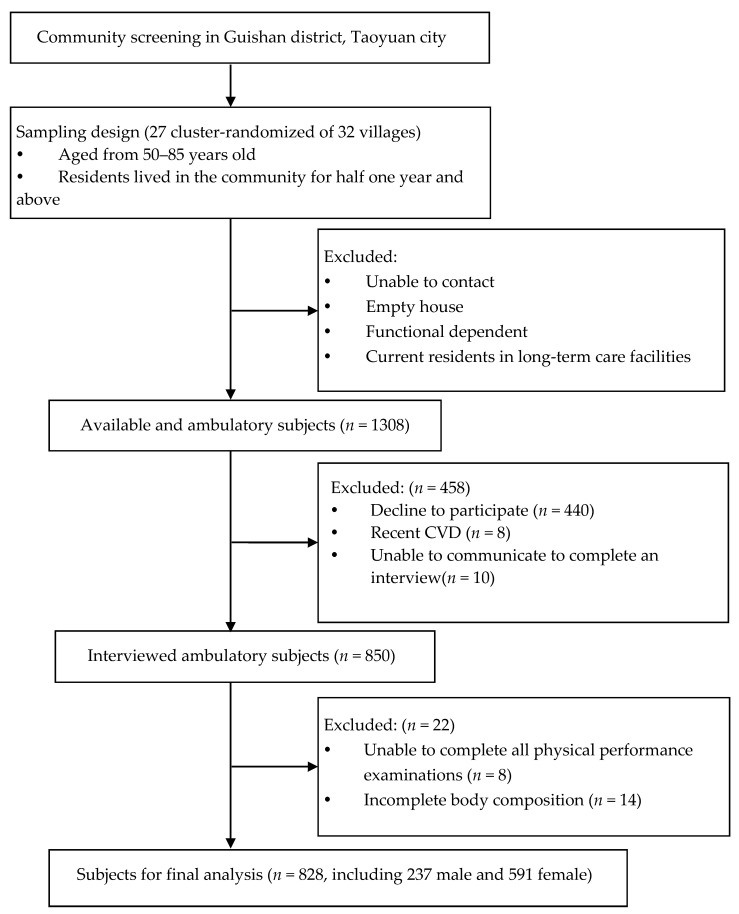
Study participant selection.

This research was a community-based and cross-sectional study, which was programmed between April and October 2017. Initially, 1308 people were recruited. The inclusion criteria were (1) age from 50 to 85 years and (2) residence in the same district for more than half a year. The exclusion criteria were (1) failure to complete body composition analysis; (2) inability to communicate adequately to complete an interview; (3) functional dependency such as inability to walk 6 m; (4) recent diagnosis of CVDs in 2 weeks; and (5) current residence in long-term care facilities. Thus, a total of 828 participants, 237 male and 591 female, were enrolled for analysis, as shown in Figure 1. The data were validated by the Institutional Review Board, and consent was obtained from all participants before enrollment.

### 2.2. Data Collection

Data collection encompassed systolic blood pressure (SBP), diastolic blood pressure (DBP), body mass index (BMI), waist circumference, body composition, gait speed, hand grip strength, exercise habit, marital status, education, past history, and frailty status. Blood pressure was checked after rest in a chair. BMI was calculated as the weight (in kg) divided by the height squared (in m^2^). Waist circumference was measured midway between the lowest ribs and the iliac crest, as recommended by the World Health Organization and International Diabetes Federation [19]. Body composition was checked with a TANITA body composition analyzer BC-418 to establish appendicular skeletal muscle mass (ASM) and appendicular skeletal mass index (ASMI). ASM was defined as the sum of the muscle mass of the four limbs, and ASMI was calculated as ASM/height^2^ (m^2^), as per the European Working Group for Sarcopenia guidelines [20]. We measured time to walk 6 m three times and calculated the average gait speed. Grip strength in both hands was measured twice with the Takei T.K.K.5401 GRIP-D handgrip dynamometer (Takei Scientific Instruments Co., Ltd., Tokyo, Japan), and we collected the best measurement. Personal information was assessed with a questionnaire administered face-to-face by a trained research assistant. Questionnaires established exercise habit, marital status, education level, and underlying diseases. Marital status featured two groups for respondents who were currently single (divorced, separated, widowed, or never married) and respondents who were currently in a couple. The participants were divided into four groups according to education level: Group 1 (uneducated), Group 2 (graduated from primary school), Group 3 (graduated from secondary school), and Group 4 (graduated from college). The underlying diseases considered were diabetes mellitus (DM), hypertension (HTN), hyperlipidemia, and CVD.

### 2.3. Assessment of Sleep Quality

Sleep quality was measured with the Chinese version of the Pittsburgh Sleep Quality Index (CPSQI). The questions of CPSQI related to habitual sleep habits during the preceding month only. The CPSQI is a reliable and valid assessment tool for use in community-based studies on poor sleep quality [21]. The cutoff point of the CPSQI was 5, which yielded high sensitivity for primary insomniacs versus controls [21]. Thus, in our study, a CPSQI score > 5 indicated poor sleep quality.

### 2.4. Definition of Frailty

To fulfill the diagnostic criteria for frailty, three of the following five components had to be satisfied: low grip strength, low energy, slow walking speed, low physical activity level, or unintentional weight loss [22]. Moreover, the definition of prefrailty satisfied one or two of five phenotypic criteria. We used two questions from the Center for Epidemiologic Studies Scale to measure low energy [23]. For the statements, “I felt that everything I did was an effort” or “I could not get going”, self-reported questionnaire answers were “occasionally” or answers indicating higher frequency. Low grip strength indicated measured grip strength of <26 kg in men or <18 kg in women. The definition of slowed walking speed was an average walking speed over six meters of <0.8 m/s. Low physical activity indicated weekly energy expenditure of less than 383 kcal for men and 270 kcal for women based on the Community Health Activities Model Program for Seniors Physical Activity Questionnaire [24]. Unintentional weight loss indicated body weight loss of >3 kg or >5% of the preceding year’s body weight.

### 2.5. Statistical Analysis

The quantitative variables used were the mean standard deviation (SD) for continuous variables and number (%) for categorical variables. Continuous variables with non-normal distributions were shown as median (interquartile range) and were calculated *p* values by Mann–Whitney U test. The independent two-sample *t* test and the chi-square test were used to calculate *p* values for continuous variables and categorical variables, respectively. Multiple logistic regression models were applied to explore the relationship between sleep quality and frailty. All statistical analyses were conducted with IBM Statistical Product and Service Solutions Statistics for Windows (version 19.0; IBM Corp., Armonk, NY, USA). Statistical significance was considered to be a *p* value of <0.05, which was corrected by false discovery rate (FDR).

## 3. Results

The results from 828 participants aged 50–85 years (28.62% male) were enrolled in this study. According to general characteristics, the study population was divided into two groups, one with CPSQI scores > 5 and one with scores ≤ 5, as shown in Table 1. Of the 828 participants, 440 (53.14%) with CPSQI scores > 5 had poor sleep quality. The proportions of participants with prefrailty or frailty status in the group with CPSQI scores ≤ 5 and the group with CPSQI scores > 5 were 21.13% and 35.00% (*p* < 0.001), respectively, suggesting that prefrailty or frailty status was more prevalent in the group with CPSQI scores > 5 than in the group with CPSQI scores ≤ 5. Moreover, SBP, ASMI, gait speed, and hand grip strength were significantly lower in the group with CPSQI scores > 5. Female, people with a low education level, and participants with HTN or hyperlipidemia had the higher proportion in the group with CPSQI scores >5. However, no significant difference was noted between two groups in age, BMI, waist circumference, exercise habit, marital status, or underlying DM or CVDs.

According to their general characteristics, members of the study population were categorized into a nonfrailty group and prefrailty/frailty group, as shown in Table 2. Of the 828 individuals, 236 (28.5%) had prefrailty or frailty status. The proportions of participants with poor sleep quality (CPSQI score > 5) increased with the severity of frailty (nonfrailty: prefrailty/frailty, 48.31%: 65.25%, *p* < 0.001), suggesting that the prefrailty/frailty group had a higher prevalence of poor sleep quality than the nonfrailty group did. In addition, participants of the prefrailty and frailty group were older. Participants with DM or CVD had the higher proportion in the prefrailty and frailty group. Gait speed and hand grip strength were lower in the prefrailty and frailty group. People of the prefrailty and frailty group had less exercise habits and lower education levels. The higher proportion of being single was seen in the prefrailty and frailty group. No significant difference was found for SBP, BMI, waist circumference, ASMI, sex, or underlying HTN or hyperlipidemia.

The results of multiple logistic regression analysis are shown in Table 3. Model 1 was adjusted for age; Model 2 was adjusted for age and sex; and Model 3 was adjusted for factors in Model 2 plus ASMI, BMI, hand grip strength, exercise habit, and gait speed. After the aforementioned confounding factors were adjusted for, poor sleep quality (CPSQI score > 5) continued to be independently associated with prefrailty or frailty status (odds ratio (OR) = 1.95, 95% confidence interval (CI) = 1.38–2.77).

## 4. Discussion

The aim of our study was to investigate the association between sleep quality and the risk of frailty. We collected the real-world data of the community in Taiwan. According to the result, poor sleep quality is independently associated with prefrailty and frailty status among middle-aged and older population members in Taiwan. This report is the first in Taiwan to use a cross-sectional study for assessing the relationship between sleep quality and prefrailty or frailty status specifically among middle-aged and older population members.

In our study, participants with lower ASMI, lower gait speed, lower hand grip strength, female, lower education level, and underlying diseases of HTN and dyslipidemia had higher proportion in the group with CPSQI scores > 5. According to previous studies, people with sarcopenia diagnosed by low ASMI, slow gait speed, and low hand grip strength had poor self-reported sleep quality [25]. Sleep complaints were relatively more prevalent in women compared to men. Poor sleep quality was reported among women with various stages of the menopause transition and post-menopause [26]. Furthermore, education level was related to insomnia, and more years of education were associated with better sleep quality [27]. Moreover, increasing the numbers of co-morbidities was associated with poor sleep quality [28]. People with poor sleep quality had increased odds of prevalent HTN and were associated with metabolic syndrome [7,29,30]. Interestingly, in our study, lower SBP was found in the group with CPSQI scores > 5. It might be supposed that participants with HTN are under medication control. Thus, the data of SBP we collected was relatively lower than the group with CPSQI scores ≤ 5. On the other hand, participants with older age, lower gait speed, lower hand grip strength, less of an exercise habit, being single, lower education level, and underlying diseases of DM and CVD had the higher proportion in the prefrailty and frailty group. As we know, older people, slow gait speed, and low hand grip strength were risk factors of frailty. Increasing physical activity or regular exercise habit were suggested to prevent for frailty. With increasing age, there is a decline in physical activity associated with decreases in exercise tolerance [31]. Thus, less of an exercise habit might be the risk of frailty. One systematic review and meta-analysis showed that unmarried individuals had a twice as high frailty risk compared to married individuals. Social factors such as living alone or social isolation were associated with frailty. The widows, the widowers, the divorced, and the separated had higher odds of frailty risks than those who never married. Those who lost their partners might experience lots of stress and might lose social support. In addition, it might decrease positive behaviors such as exercise habits [32]. People with a lower education level might have less of a concept of regular exercise and have not enough financial resources or time to do it. Thus, it might increase the risk of frailty. Previous studies showed that muscle strength and quality would decrease in people with DM due to insulin resistance and chronic inflammation. The dysregulation of levels of various hormones and nutrition was described in people with insulin resistance [33]. These would be the risks of frailty. People with CVD would decrease the physical activities, and that is why it would be associated with frailty [34]. Although the relationship between sleep quality and frailty had been discussed, in our study, not only the elderly but also middle-aged population was enrolled; this strength is in good agreement with the notion that middle-aged individuals should be included for the study of identification and prevention of frailty [18]. In addition, our community-based and real-world data widely involved participants with various health statuses. Thus, our data are different from those collected in hospitals, which might have selective bias because non-symptomatic people would not be included.

In our study, poor sleep quality is independently associated with prefrailty and frailty in the community population. In previous research, sleep disorders elevated the risk of CVDs, metabolic diseases, neurogenic diseases, and psychological diseases [7,35]. This is one possible mechanism through which multi-comorbidities related to sleep problems cause poor health status, which would increase the risk of being frail. In addition, sleep disturbance influenced inflammatory regulation and sleep loss, short sleep duration, and complaints of sleep disturbance were associated with increases in inflammation [36]. Moreover, sleep disturbance has demonstrated some connection with frailty in elderly people. One systematic review of six cross-sectional studies revealed an association between sleep disturbance and frailty among elderly people [15]. One systemic review and meta-analysis showed abnormal sleep duration to be associated with an increased risk of frailty among elderly individuals [16]. Other research showed that oxidative stress and inflammation were potential drivers of frailty [37]. Although the pathogenesis between sleep disturbance and frailty is unclear, one possibility, according to the research, is that inflammatory pathways are influenced by the sleep–wake disturbance, which affects the development of frailty.

Frailty causes poor prognosis in elderly people. Three of the five Fried frailty index indicators (slow gait speed, low physical activity, and unintentional weight loss) were independently associated with chronic disability, long-term nursing home stays, and death [38]. In particular, slow gait speed was found to be a predictor of chronic disability (hazard ratio (HR) = 2.97, 95% CI = 2.32–3.80), long-term nursing home care (HR = 3.86, 95% CI = 2.23–6.67) and injurious falls (HR = 2.19, 95% CI = 1.33–3.60) [38]. Furthermore, prefrailty status fulfilling only one or two criteria of frailty also increased the risk of frailty [11]. One study showed some factors including high psychological distress, living alone, having health worries, and poor sleep quality; stair climbing, appetite, hydration; continence, and total food intake might be the predictive capacities for prefrailty to frailty [39]. With increasing age, there is also a decline in physical activity associated with decreases in exercise tolerance, which would lead to an increasing risk of frailty [31]. Thus, early prevention of prefrailty and frailty status is a concern for everyone, particularly elderly people.

Frailty is defined as an age-related syndrome [11]. Aging is a major risk factor for frailty. Women have an increased risk of sleep disturbance across their lifespans compared with men [40]. During times of hormonal changes in women, sleep regulation and arousals are affected, particularly in the menopausal transition and in early postmenopause [40,41]. The average age of menopause in Taiwan is 50–54 years [42]. Participants at the age of menopausal transition and early postmenopause were both enrolled in our study. Therefore, we had to remove these confounding factors before discussing the relationship between poor sleep quality and frailty status. After adjusting for variables with known or suspected impacts on prefrailty and frailty status, poor sleep quality continued to be significantly associated with prefrailty and frailty status in our study. In addition to traditional risk factors for frailty, according to one population-based cohort study, social and behavioral factors including education and marital status were associated with frailty [43]. Notably, this study showed that associations were present among those with an education level lower than high school (OR = 1.57, 95% CI = 1.12–2.22) and among those living with families (versus with spouses; OR = 1.76, 95% CI = 1.05–2.94) [43]. Similar results were observed in our study.

Our study is the first cross-sectional study to investigate the relationship between sleep quality and frailty status in middle-aged and older Taiwanese people. However, some limitations apply. First, the study is cross-sectional and therefore, it is unable to describe the causal relationship between sleep quality and prefrailty or frailty status. Second, because the data collection was community-based, the participants came only from Northern Taiwan, increasing the uncertainty of the ecological validity of the findings and the possibility of healthy volunteer bias. Thus, the results of our study should not be extrapolated to other regions in Taiwan. A potential healthy volunteer bias might have lowered the prevalence of prefrailty and frailty status and decreased our capabilities to control for confounding. Third, approximately 70% of the participants were female, and this would have caused the prevalence of poor sleep quality to be overestimated, because sleep disturbance is more prevalent for women than men [44]. Fourth, prefrailty and frailty status were not discussed separately in our study, and it might be our further vision that we could investigate in the future. Fifth, the information of participants with menopause was insufficient in our study. This could be the further vision for us to investigate in the future. Finally, frailty risk might be increased not only by the aforementioned diseases but also by diseases such as neurologic disorders. More participants with multiple chronic conditions should be enrolled in further studies.

## 5. Conclusions

Our study suggests that poor sleep quality is independently associated with prefrailty and frailty status among middle-aged and older adults in Taiwan. Therefore, sleep quality should be further assessed for frailty status among middle-aged and older adults, but the effects of sleep quality on frailty incidence require further validation in longitudinal studies.

## Figures and Tables

**Table 1:** General characteristics of the study population according to Chinese version of the Pittsburgh Sleep Quality Index (CPSQI) score.

Variables	CPSQI			
	Total (*n* = 828)	CPSQI ≤ 5 (*n* = 388)	CPSQI > 5 (*n* = 440)	*p* Value
Age (year) ^¶^	64.00 (59.00, 70.00)	64.00 (59.00, 69.00)	65.00 (60.00, 71.00)	0.21
SBP (mmHg) ^¶^	127.00 (116.00, 138.00)	128.00 (118.00, 139.00)	125.50 (115.00, 136.75)	0.047 *
BMI (kg/m^2^) ^¶^	24.20 (22.00, 26.60)	24.35 (22.10, 26.70)	24.20 (21.93, 26.60)	0.75
WC (cm)	85.08 ± 10.07	85.56 ± 9.82	84.67 ± 10.28	0.20
ASMI (kg/m^2^) ^¶^	7.07 (6.55, 8.05)	7.21 (6.58, 8.25)	6.97 (6.52, 7.89)	0.02 *
Gait speed (m/s) ^¶^	1.43 (1.30, 1.58)	1.46 (1.29, 1.61)	1.41 (1.26, 1.56)	0.02 *
Hand grip strength (kg) ^¶^	25.60 (21.70, 31.68)	26.50 (21.80, 33.85)	24.80 (21.43, 29.70)	0.002 ^§^
Men, *n* (%)	237 (28.62%)	126 (32.47%)	111 (25.23%)	0.02 *
Exercise habit, *n* (%)	602 (72.71%)	282 (72.68%)	320 (72.73%)	0.99
Marital status (single), *n* (%)	171 (20.65%)	70 (18.04%)	101 (22.95%)	0.08
Education level				0.03 *
No, *n* (%)	65 (7.85%)	27 (6.96%)	38 (8.64%)	
Primary, *n* (%)	314 (37.92%)	132 (34.02%)	182 (41.36%)	
Secondary, *n* (%)	366 (44.20%)	181 (46.65%)	185 (42.05%)	
College, *n* (%)	83 (10.02%)	48 (12.37%)	35 (7.95%)	
DM, *n* (%)	119 (14.37%)	51 (13.14%)	68 (15.45%)	0.34
HTN, *n* (%)	265 (32.00%)	107 (27.58%)	158 (35.91%)	0.01 *
Hyperlipidemia, *n* (%)	111 (13.41%)	41 (10.57%)	70 (15.91%)	0.02 *
CVD, *n* (%)	63 (7.61%)	22 (5.67%)	41 (9.32%)	0.05
Frailty status				<0.001 ^§^
Non-frailty, *n* (%)	592 (71.50%)	306 (78.87%)	286 (65.00%)	
Pre-frailty/frailty, *n* (%)	236 (28.50%)	82 (21.13%)	154 (35.00%)	

Notes: Clinical characteristics are expressed as mean ± SD values for continuous variables and *n* (%) for categorical variables. ^¶^ Continuous variables with non-normal distributions are shown as median (interquartile range). *p* values were derived from the independent two-sample *t* test and Mann–Whitney U test for continuous variables and the chi-square test for categorical variables. * *p* value < 0.05; ^§^ *p* value < (0.05/16). Abbreviations: SBP, systolic blood pressure; BMI, body mass index; WC, waist circumference; ASMI, appendicular skeletal muscle index; DM, diabetes mellitus; HTN, hypertension; CVD, cardiovascular disease.

**Table 2:** General characteristics of the study population according to frailty status.

Variables	Frailty			
	Total	Non-Frailty	Prefrailty/Frailty	*p* Value
	(*n* = 828)	(*n* = 592)	(*n* = 236)	
Age (year) ^¶^	64.00 (59.00, 70.00)	64.00 (59.00, 69.00)	66.00 (60.00, 73.00)	<0.001 ^§^
SBP (mmHg) ^¶^	127.00 (116.00, 138.00)	127.00 (116.00, 138.00)	125.00 (116.25, 137.75)	0.56
BMI (kg/m^2^) ^¶^	24.20 (22.00, 26.60)	24.20 (22.10, 26.60)	24.30 (21.80, 26.68)	0.98
WC (cm)	85.08 ± 10.07	84.72 ± 9.65	86.00 ± 11.01	0.10
ASMI (kg/m^2^) ^¶^	7.07 (6.55, 8.05)	7.06 (6.55, 8.06)	7.14 (6.56, 8.06)	0.38
Gait speed (m/s) ^¶^	1.43 (1.30, 1.58)	1.46 (1.31, 1.61)	1.34 (1.16, 1.54)	<0.001 ^§^
Hand grip strength (kg) ^¶^	25.60 (21.70, 31.68)	26.50 (23.00, 32.80)	23.00 (17.60, 29.20)	<0.001 ^§^
Men, *n* (%)	237 (28.62%)	166 (28.04%)	71 (30.08%)	0.56
Exercise habit, *n* (%)	602 (72.71%)	450 (76.01%)	152 (64.41%)	0.001 ^§^
Marital status (single), *n* (%)	171 (20.65%)	109 (18.41%)	62 (26.27%)	0.01 *
Education level				0.001 ^§^
No, *n* (%)	65 (7.85%)	35 (5.91%)	30 (12.71%)	
Primary, *n* (%)	314 (37.92%)	215 (36.32%)	99 (41.95%)	
Secondary, *n* (%)	366 (44.20%)	276 (46.62%)	90 (38.14%)	
College, *n* (%)	83 (10.02%)	66 (11.15%)	17 (7.20%)	
DM, *n* (%)	119 (14.37%)	74 (12.50%)	45 (19.07%)	0.02 *
HTN, *n* (%)	265 (32.00%)	181 (30.57%)	84 (35.59%)	0.16
Hyperlipidemia, *n* (%)	111 (13.41%)	87 (14.70%)	24 (10.17%)	0.08
CVD, *n* (%)	63 (7.61%)	38 (6.42%)	25 (10.59%)	0.04 *
Sleep quality				<0.001 ^§^
CPSQI ≦ 5, *n* (%)	388 (46.86%)	306 (51.69%)	82 (34.75%)	
CPSQI > 5, *n* (%)	440 (53.14%)	286 (48.31%)	154 (65.25%)	

Notes: Clinical characteristics are expressed as mean ± SD values for continuous variables and as *n* (%) for categorical variables. ^¶^ Continuous variables with non-normal distributions are shown as median (interquartile range). *p* values were derived from the independent two-sample *t* test and Mann–Whitney U test for continuous variables and from the chi-square test for categorical variables. * *p* value < 0.05; ^§^ *p* value < (0.05/16). Abbreviations: SBP, systolic blood pressure; BMI, body mass index; WC, waist circumference; ASMI, appendicular skeletal muscle index; DM, diabetes mellitus; HTN, hypertension; CVD, cardiovascular disease.

**Table 3:** Association between sleep quality and frailty status by multiple logistic regression analysis.

Variables	Model 1			Model 2			Model 3		
	OR	95% CI	*p* Value	OR	95% CI	*p* Value	OR	95% CI	*p* Value
CPSQI ≤ 5	1	-	-	1	-	-	1	-	-
CPSQI > 5	2.00	(1.46–2.75)	<0.001	2.01	(1.46–2.76)	<0.001	1.95	(1.38–2.77)	<0.001

Notes: Model 1: Multiple logistic regression adjusted for age. Model 2: Multiple logistic regression adjusted for age and sex. Model 3: Multiple logistic regression adjusted for factors in Model 2 plus ASMI, BMI, hand grip strength, exercise habit, and gait speed. Abbreviations: CI: confidence interval; OR: odds ratio.
